# Impact of concentrated growth factor (CGF) injection on acceleration of orthodontic tooth movement in rabbits

**DOI:** 10.1186/s12903-024-04520-2

**Published:** 2024-07-12

**Authors:** Safa B. Alawy, Mona Al Ahmady El Meligy, Eman M. Salem, Wafaa Yahia Alghonemy

**Affiliations:** 1 Orthodontics, Faculty of Dentistry, Tanta University, Tanta, Egypt; 2 Oral Medicine, Periodontology, Oral Diagnosis and Oral Radiology Faculty of Dentistry, Tanta University, Tanta, Egypt; 3 Oral Biology, Faculty of Dentistry, Pharos University in Alexandria, P. O. Box 37, Sidi Gaber, Alexandria, Egypt; 4 Department of Basic Medical and Dental Sciences, Faculty of Dentistry, Zarqa University, Zarqa, 13110 Jordan; 5 Oral Biology Department, Faculty of Dentistry, Tanta University, Tanta, 31527 Egypt

**Keywords:** Orthodontic tooth movement, Concentrated growth factor, Accelerated orthodontics, Growth factors

## Abstract

**Background:**

The present study aimed to assess how a concentrated growth factor (CGF) injection affects the rate of orthodontic tooth movement in rabbits.

**Methods:**

This experimental investigation employed a split-mouth configuration. Before orthodontic mesialization of the maxillary first molars, CGF was prepared and administered using submucosal injections on the buccal and palatal sides of the maxillary first molars in one randomly assigned quadrant. The opposite quadrant was used as a control. The study examined four time points:1, 2, 3, and 4 weeks. The measurement of tooth movement was conducted at each follow-up point using a digital caliper. The rabbits were euthanized, and their maxillary segments, specifically the maxillary first molars, were studied histologically to identify any alterations occurring on both the tension and compression sides.

**Results:**

Significant tooth movement was observed in the experimental sides versus control sides in the second, third, and fourth week of follow-up periods (*p* ≤ 0.05). Histologically, on the compression side, the CGF group showed bone resorption and periodontal ligament active reactions from the first week and continued throughout the next three weeks. Also, on the tension side, the CGF group depicted cementoblastic and osteoblastic activities from the first week followed by fibroblastic activities from the second week and all activities continued till the fourth week.

**Conclusions:**

CGF has the potential to effectively enhance orthodontic tooth movement without adverse clinical or histological effects.

## Background

The duration of orthodontic treatment is crucial in determining patient satisfaction, and there seems to be a growing demand for shorter treatment durations. Orthodontic therapy can vary from a few months to several years. However, most thorough treatments typically last approximately 24 months to finish. However, the treatment duration may be extended in cases of severe malocclusion [1, 2].

During lengthy treatments, patients are more likely to experience root resorption, gingival hyperplasia, and white spot lesions. Moreover, prolonged treatment time may lead to incompliance among adolescent patients, making case completion more challenging [3–6].

Prior research has examined the use of pharmacological substances such as prostaglandin or hormones like parathyroid hormone to accelerate orthodontic tooth movement (OTM) [7, 8]. Nevertheless, these techniques are biochemically based, demanding in terms of preparation and application, and using supplementary hormones can lead to unfavorable systemic consequences [9–11]. Physical ways are costly since they require special devices to perform them [12–14]. While surgical techniques are safe and effective, they are also invasive and may not be acceptable to most patients [15, 16]. Flapless micro-osteoperforation was recently introduced, yet contradictory findings were found in the literature regarding this method [17–20].

Growth Factors (GF) are proteins that control the intricate processes involved in wound healing. They are also crucial in angiogenesis, cell migration, proliferation, and synthetic induction of bone cells, all affecting physiological bone remodeling and repair [21]. Numerous growth factors are involved in the repair of bones; however, two significant groups may be distinguished: growth factors produced from bone and growth factors obtained from autologous blood that are released when platelets are activated [22]. To hasten wound healing and repair, adjuvant agents comprising platelet concentrates (PCs), such as platelet-rich plasma (PRP), platelet-rich fibrin (PRF), and concentrated growth factor (CGF), may be utilized [23].

In 2006, Sacco made the initial proposal for the CGF [24]. After platelet-rich plasma and fibrin, this blood extract represents the third generation. CGF is an organic matrix that is rich in fibrin and comprises growth factors, leukocytes, platelets, immunological cells, and CD34 + stem cells. These components play a vital role in the process of regeneration. CGF is a biologically active substance that promotes bone development and tissue healing [25, 26].

Compared to early-generation platelet concentrates like PRF, more growth factors, increased viscosity, increased tensile strength, and increased adhesive strength are all present in CGF [22]. Together with these, it releases other GFs that promote angiogenesis, matrix remodeling, and cell proliferation, including fibroblast growth factor (FGF), vascular endothelial growth factor (VEGF), brain-derived growth factor (BDGF), platelet-derived growth factor (PDGF), transforming growth factor-β1 (TGF-β1) and β2 (TGF-β2), and insulin-like growth factor (IGF). It increases the vascular endothelial growth factor content and facilitates soft tissue healing since it incorporates many GFs [23, 26].

CGF in orthodontics can potentially aid tooth movement by promoting accelerated healing and tissue regeneration by enhancing the growth of new blood vessels and improving tissue regeneration, which can be beneficial during orthodontic procedures and expedite recovery [27]. Moreover, The multitude of growth factors in CGF may be beneficial in promoting the action of osteoblasts and osteoclasts that are linked to OTM [28, 29].

From this standpoint, this study aimed to assess how a CGF injection affects the rate of orthodontic tooth movement in New Zealand white rabbits.

## Methods

The present investigation was conducted in adherence to the protocols established by the Research Ethics Committee of the Faculty of Dentistry, Tanta University, under ethical code #R-ORTH-3-23-4.

### Sample size calculation

The smallest sample size was determined using data from a prior study that examined the effects of injecting PRP against PRF in rabbits [30]. Adopting power of 80% (β = 0.20) to detect a standardized effect size in OTM (primary outcome) of 0.8140, and significance level 5% (accepted α error = 0.05), the negligible necessary sample size was considered to be six sides per subgroup (number of groups = 2) (number of time points = 4) (Total sample size = 6 *2 * 4 = 48 sides) [31]. As split mouth is the adopted design. Thus, 24 New Zealand white rabbits (6 per group) were considered the total number. Any specimen loss resulting from processing error will be substituted to uphold the sa`mple size [32].

### Group assignment and animal preparation

Twenty-four adult New Zealand white rabbits (with 48 maxillary quadrants), 7–10 months old, and nearly 2–3 kg weight with normal dentition. Rabbits were obtained from Faculty of Agriculture, Alexandria University. They were selected two weeks before the experiment and examined for any general or dental diseases for exclusion. The animals received food and water and were kept in an environment with controlled lighting and temperature throughout the experimental period, according to the declaration of Helsinki [33]. The 48 maxillary quadrants were used equally in the experimental and control sides and then subdivided according to the follow-up periods into four sub-groups, as presented in Table 1. The surgery was managed under general anesthesia, where 0. 4 ml/Kg of atropine sulfate was injected intramuscularly as a premedication drug, followed by an injection of a mixture of ketamine hydrochloride 10% (ketamine alfasan10%, Woerden, The Netherlands) and xylazine 2% (Adwia, 10th of Ramadan City, Egypt). At a dose of 0.5,0.2 ml/Kg body weight, respectively,

**Table 1:** Animal grouping as used in the study

Groups	Subgroups
Group 1 (Experimental): (*n* = 24 sides)	**Subgroup A (*n*** **= 6)**: Follow-up for one week
	**Subgroup B (*n*** **= 6)**: Follow-up for two weeks
	**Subgroup C (*n*** **= 6)**: Follow-up for three weeks
	**Subgroup D (*n*** **= 6)**: Follow-up for four weeks
Group 2 (Control): (*n* = 24 sides)	**Subgroup A (***n* **= 6)**: Follow-up for one week
	**Subgroup B (***n* **= 6)**: Follow-up for two weeks
	**Subgroup C (***n* **= 6)**: Follow-up for three weeks
	**Subgroup D (***n* **= 6)**: Follow-up for four weeks

### CGF preparation and injection

**Fig. 1:**
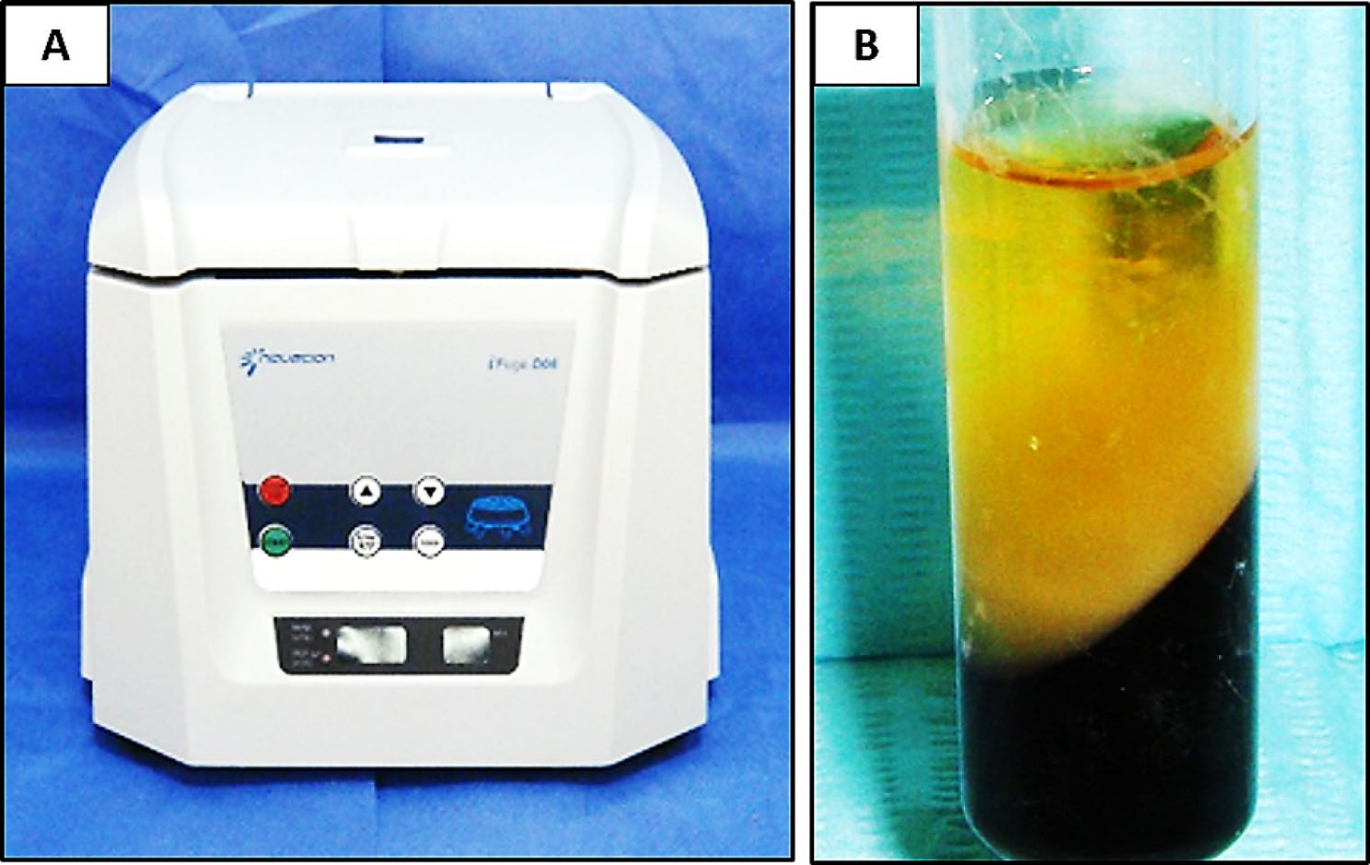
(**A**) showing the centrifuge used in the procedure, (**B**) showing the prepared CGF

Ten milliliters of rabbit blood were extracted, and tubes were stored for the one-step centrifugation procedure (NEUATION IFUGE D06) [34] (Fig. 1a): 30 s for acceleration, 2 min for 2700 rpm, 4 min for 2400 rpm, 3 min for 3000 rpm, and 36 s for deceleration and stop. This leads to the following four phases [35]: (Fig. 1b).

1. The superior phase is symbolized by serum.
2. A fibrin buffy coat represents the transitional stage.
3. Growth factors represent the liquid phase.
4. The lower phase is represented by red blood cells.

Utilizing an insulin syringe, seventy units of the liquid phase was administered submucosally into the buccal and palatal regions across from the maxillary first molars in the same manner that the local anesthetic injection was done on all experimental sides [36].

### Orthodontic procedures

A simple orthodontic appliance was installed on each maxillary quadrant using a NiTi closed coil spring (9 mm, DynaFlex, USA, www.dynaflex.com). The spring is attached from each side to the ligature wire. These ligature wires were then ligated to holes generated using rosehead burs in the upper first molars and the cervical portion of central incisors. The force-gauge measurement indicated that the spring stretched to deliver 40 cN force [37]. The ends of the ligature wires were covered by flowable composite to prevent gingival irritation. The springs were not reactivated between the follow-up periods.

A digital caliper (Vernier Digital Caliper, Future Electronics, Egypt) was used to measure how far the first molars were mesialized on both the experimental and control sides by measuring the distance between two reference points: the mesial contact point of the first molars to the distal contact point of the central incisors. All measurements were done by the same investigator who was blinded to the treatment groups.

### Animal’s euthanization

Six rabbits were euthanized after 1, 2, 3, and 4 weeks, respectively. The rabbits authenization was done by intramuscular injection of overdose of xylazine HCl (30 mg/kg; Adwia, 10th of Ramadan City, Egypt) and ketamine HCl (70 mg/kg; ketamine alfasan10%, Woerden, The Netherlands) and scarified then dissected.

### Histologic evaluation

The maxilla of each rabbit was dissected into right and left halves, coded, and fixed into 10% calcium formol (Aqua Med Company, Egypt) for 48 h. After that, they were washed and decalcified through immersion in EDTA 10%. Then, the samples underwent progressively higher alcohol concentrations for dehydration before being conventionally embedded in paraffin. Hematoxylin and eosin (H&E) were used to stain six µm thick sagittal serial sections for histological analysis under a light microscope (Leica ICC50 HD) outfitted with a digital camera, and images of representative regions were captured and annotated [38]. PDL, alveolar bone, and the root of the upper first molars were histologically observed from the mesial (compression side) and distal (tension side). Histological analysis was done by a single investigator in a blinded manner.

### Statistical analysis

All data were statistically analyzed using version 20.0 of the IBM SPSS software package (Armonk, NY: IBM Corp). The Shapiro-Wilk test was used to check for normality in continuous data. Distributed data were represented by mean, standard deviation, range, and median. A paired t-test was employed to compare two periods for normally distributed quantitative variables. A one-way ANOVA test was performed to compare the different studied periods, and a post hoc Tukey test was performed for pairwise comparisons. The obtained results were declared significant at the 5% level. All data were labelled with numbers and the statistician was also blinded regarding the study groups.

## Results

All animals survived the experimental procedures and the subsequent follow-up phase. No inflammation-related symptoms were noticed. Only 20% of the animals experienced gingival edema related to coil springs, which eventually resolved within a few days.

### Tooth movement rate

**Fig. 2:**
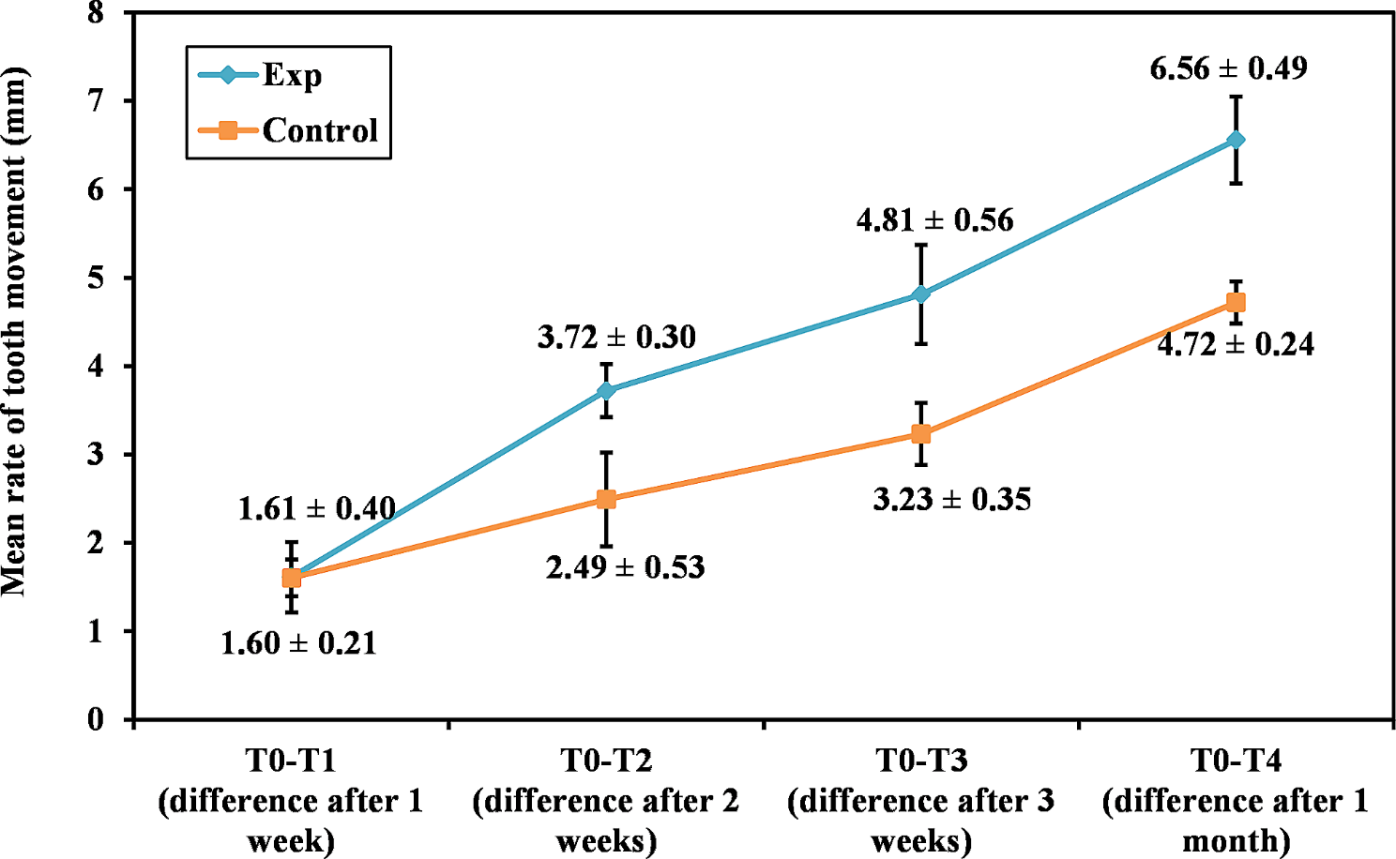
Experimental versus control groups concerning the tooth movement (mm) rate at different follow-up periods

The distance measured between the first molars and incisors was decreased throughout follow-ups in both the experimental and control sides. However, the CGF results in a higher rate of tooth movement starting from the second to the fourth weeks compared to the control sides (3.72 ± 0.30 vs. 2.49 ± 0.53, 4.81 ± 0.56 vs. 3.23 ± 0.35, and 6.56 ± 0.49 vs. 4.72 ± 0.24, respectively). According to the Paired t-test results, a significant difference between the experimental and control sides was demonstrated in all examined periods (*p* ≤ 0.05) except for the first week (*p* = 0.951). (Table 2; Fig. 2)

**Table 2:** Comparative analysis of different studied groups concerning tooth movement (mm) rate at different follow-up periods

Follow up periods	Experimental (*n* = 6)	Control (*n* = 6)	t	*p*
T0-T1 (difference after one week)				
Mean ± SD	1.61 ± 0.40	1.60 ± 0.21	0.064	0.951
Median (Min. – Max.)	1.48 (1.2–2.24)	1.55 (1.39–1.97)		
T0-T2 (difference after two weeks)				
Mean ± SD	3.72 ± 0.30	2.49 ± 0.53	5.563^*^	0.003^*^
Median (Min. – Max.)	3.57 (3.47–4.16)	2.71 (1.44–2.83)		
T0-T3 (difference after three weeks)				
Mean ± SD	4.81 ± 0.56	3.23 ± 0.35	6.358^*^	0.001^*^
Median (Min. – Max.)	4.58 (4.26–5.80)	3.21 (2.68–3.74)		
T0-T4 (difference after one month)				
Mean ± SD	6.56 ± 0.49	4.72 ± 0.24	6.218^*^	0.002^*^
Media (Min. – Max.)	6.50 (6.10–7.44)	4.78 (4.35–4.99)		

SD is for standard deviation, while t stands for paired t-test

p: p value for comparison between **Exp** and **Control** in each period

*: Statistically significant at *p* ≤ 0.05

There was a significant difference between all follow-up weeks, according to the One-Way ANOVA test and the post-hoc test (Tukey) used for comparing the various studied periods of each group. (Table 3)

**Table 3:** Comparative analysis of different studied periods concerning each group’s tooth movement (mm) rate

Group	T0-T1	T0-T2	T0-T3	T0-T4	F	*p*
Exp (*n* = 6)						
Mean ± SD	1.61 ± 0.40	3.72 ± 0.30	4.81 ± 0.56	6.56 ± 0.49	126.489^*^	< 0.001^*^
Median (Min. – Max.)	1.48 (1.2–2.24)	3.57(3.47– 4.16)	4.58(4.26–5.80)	6.5 (6.1–7.44)		
Sig. bet. periods	p_1_ < 0.001^*^, p_2_ < 0.001^*^, p_3_ < 0.001^*^, p_4_ = 0.002^*^, p_5_ < 0.001^*^, p_6_ < 0.001^*^					
Control (*n* = 6)						
Mean ± SD	1.60 ± 0.21	2.49 ± 0.53	3.23 ± 0.35	4.72 ± 0.24	84.204^*^	< 0.001^*^
Median (Min. – Max.)	1.55(1.39–1.97)	2.71(1.44– 2.83)	3.21(2.68–3.74)	4.78(4.35– 4.99)		
Sig. bet. periods	p_1_ = 0.001^*^, p_2_ < 0.001^*^, p_3_ < 0.001^*^, p_4_ = 0.009^*^, p_5_ < 0.001^*^, p_6_ < 0.001^*^					

SD: Standard deviation

F: F for One way ANOVA test, pairwise comparison between each two groups was done using a Post Hoc Test (Tukey)

p_1_: p-value comparing the first and the second weeks

p_2_: p-value comparing the first and the third weeks

P3: p-value comparing the first and the fourth weeks

p_4_: p-value comparing the second and the third weeks

P5: p-value comparing the second and the fourth weeks

p_6_: p-value comparing the third and the fourth weeks

*: Significance at *p* ≤ 0.05

### Histologic findings

#### At the compression side

**Fig. 3:**
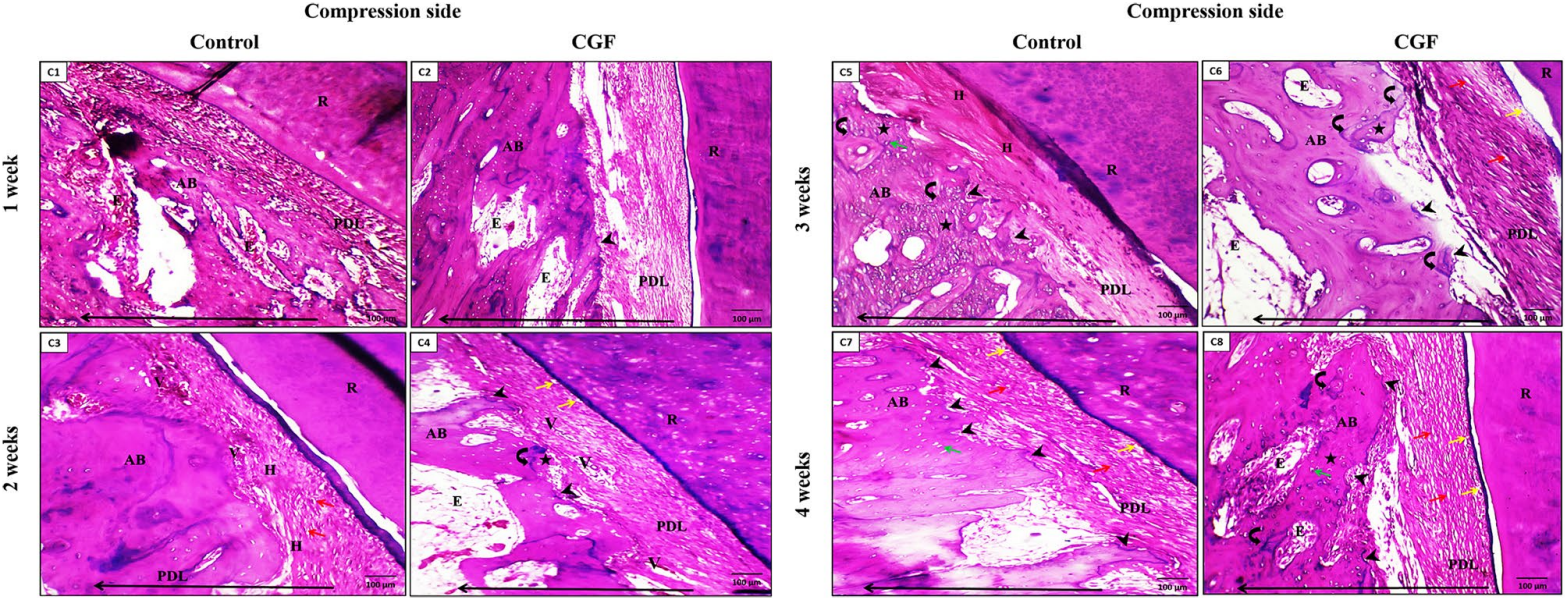
Photomicrographs of the **compressive side** of both groups. (**C1& C2**) after **one week** in control and CGF groups, respectively, showing compressed PDL. C2, the CGF group, shows the initiation of bone resorption (arrowhead) at the bone surface. (**C3**) control group after **two weeks** showing PDL fibers with obvious hyalinization (H) and large active fibroblasts (red arrows). (**C4**) CGF group after two weeks showing PDL fibers with dilated blood vessels (V), active cementoblasts (yellow arrows), and areas of bone resorption (arrowheads) at the bone surface. Also, a large area of new bone deposition (star) is delineated from the old one with dense appositional lines (curved arrows). (**C5**) The control group showed PDL fibers with obvious hyalinization (H) and areas of bone resorption (arrowheads) at the bone surface at three weeks. The new woven bone (stars) is delineated from the old one with dense appositional lines (curved arrows) with irregularly arranged osteocytes (green arrows). (**C6**) CGF group at three weeks showing PDL fibers with noticeable large active fibroblasts (red arrows), active cementoblasts (yellow arrows), and multiple areas of bone resorption (arrowheads) at the bone surface. Also, areas of new bone deposition (stars) are delineated from old bone with appositional lines (curved arrows), and large multiple areas of extravasated blood in the bone (E) could be detected. (**C7**) The control group, after **four weeks**, showed PDL fibers with noticeable large active fibroblasts (red arrows), cementoblastic activity (yellow arrows), and multiple areas of bone resorption (arrowheads) at the bone surface. The woven bone fills the socket (AB) with irregularly arranged osteocytes (green arrow). (**C8**) CGF group after four weeks of orthodontic force application showing PDL fibers with noticeable large active fibroblasts (red arrows), active cementoblasts (yellow arrows), and multiple areas of bone resorption (arrowheads) at the bone surface. The woven bone fills the socket (AB). It shows irregularly arranged osteocytes (green arrow), an area of new bone deposition (star) that is delineated from the old one with dark lines (curved arrows), and large multiple areas of extravasated blood (E). Notice the long arrow that indicates the direction of the tooth movement at the bottom of each figure. (H&E stain, C1, 2, 3, 4, 5, 6, 7, 8 × 100)

**In the control group**, the PDL appeared compressed after one week of orthodontic force application, followed by PDL hyalinization in different areas and fibroblastic activity after two weeks of initial orthodontic application (Fig. 3C1,3). Moreover, in week 3, the PDL increased hyalinization and bone resorption, and irregularly arranged osteocytes in the woven bone appeared (Fig. 3C5). PDL showed the same changes at four weeks as the previous week, with three plus cementoblastic and fibroblastic activities (Fig. 3C7).

**In the CGF group**, the PDL appeared compressed after one week of initial orthodontic force application, plus the initiation of bone resorption at the bone surface was depicted (Fig. 3C2). After two weeks of orthodontic treatment, PDL fibers showed dilated Blood vessels, active cementoblasts, and areas of bone resorption at the bone surface (Fig. 3C4). The PDL showed the same histological changes as the previous interval plus noticeable large active fibroblasts and irregularly arranged large osteocytes in the woven bone that filled the socket in weeks 3 and 4 (Fig. 3C6, 8)—compared with the control group, principal fibers of PDL and fibroblasts displayed a more regular pattern of organization devoid of inflammatory infiltrates or hyaline zones.

#### At the tension side

**Fig. 4:**
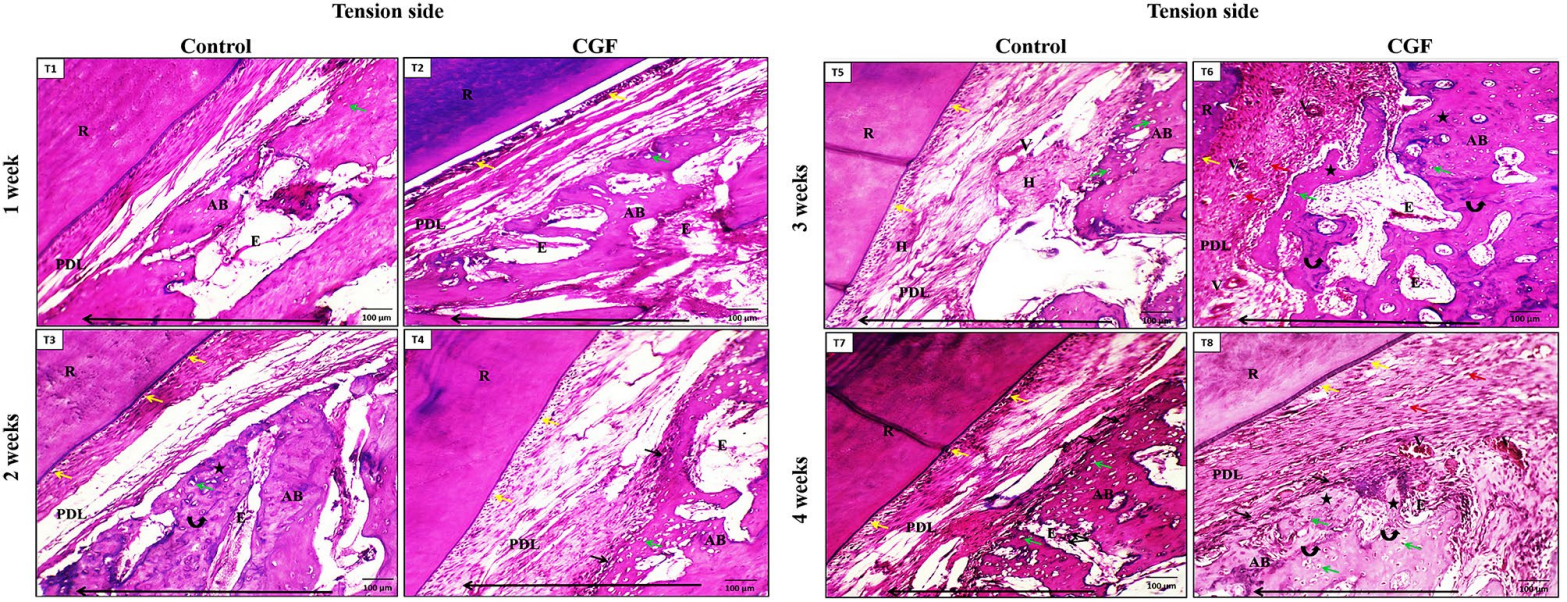
Photomicrographs of the **tension side** of both groups. (**T1**) The control group, after one week, showed tensed PDL and bone filling the socket (AB) with a small number of large osteocytes (green arrow). (**T2**) CGF group after one week showing tensed PDL, cementoblastic activity (yellow arrows), and the woven bone filling the socket (AB) with irregularly arranged large osteocytes (green arrow) as well as extravasated red blood cells (E) scattered throughout the alveolar bone. (**T3**) Control group at **two weeks** showing cementoblastic activity (yellow arrows), irregularly arranged large osteocytes (green arrow) occupying the newly formed woven bone of the socket (star) that delineated from the old one with dark lines (curved arrows). Also, extravasated red blood cells (E) at random areas of the bone could be detected. (**T4**) CGF group after **two weeks** showing cementoblastic activity (yellow arrows) The woven bone is filling the socket (AB) with irregularly arranged large osteocytes (green arrow) and active osteoblasts (black arrows). Also, extravasated red blood cells (E) scattered throughout the alveolar bone could be noticed. (**T5**) The control group showed PDL fibers after three weeks, with areas of hyalinization (H) and dilated blood vessels (V). The woven bone fills the socket (AB) with irregularly arranged large osteocytes (green arrow). (**T6**) CGF group after four weeks showing PDL fibers with dilated blood vessels (V), large active fibroblasts (red arrows), and cementoblastic activity (yellow arrows). The woven bone fills the socket (AB) with new bone formation (stars) separated from old bone with appositional lines (curved arrows). It contains irregularly arranged osteocytes (green arrow) and extravasated blood (E) areas. Notice the large cementocytes inside the root cementum (white arrows). (**T7**) The control group showed PDL fibers with cementoblastic activity at four weeks (yellow arrows). The woven bone fills the socket (AB) with irregularly arranged large osteocytes (green arrow) and osteoblastic activity (black arrows). Notice the extravasated blood (E) areas with inflammatory infiltrates (branched arrow). (**T8**) CGF group after three weeks showing PDL fibers with dilated Blood vessels (V), large active fibroblasts (red arrows), and cementoblastic activity (yellow arrows). The woven bone fills the socket (AB) with an area of newly formed bone (star) that is delineated from old bone with appositional lines (curved arrows), irregularly arranged large osteocytes (green arrow), extravasated blood vessels (E), and osteoblastic activity (black arrows). Notice the long arrow that indicates the direction of the tooth movement at the bottom of each figure. (H&E stain, T1, 2, 3, 4, 5, 6, 7, 8 × 100)

**In the control group**, The PDL appeared tense, and some small osteocytes appeared in the bone of the socket after one week of orthodontic force application (Fig. 4T1). At 2-week intervals, the tensed PDL showed cementoblastic activity and irregularly arranged large osteocytes occupying the woven bone of the socket (Fig. 4T3). Besides the irregularly arranged large osteocytes in the woven bone and the dilated blood vessels, areas of hyalinization appeared after 3-week intervals (Fig. 4T5). Also, at 4-week intervals, the PDL of the control group showed cementoblastic activity and woven bone osteoblastic activity (Fig. 4T7).

**In the CGF group**, The PDL appeared tensed, and cementoblastic activity was detected. Also, the woven bone with irregularly arranged large osteocytes was noticed after one week of orthodontic force application (Fig. 4T2). At a 2-week interval, the PDL and the bone showed the same changes as the previous interval, plus cementoblastic and osteoblastic activities (Fig. 4T4). At 3-week intervals, the PDL and the woven bone showed the same changes as the previous intervals, plus dilated blood vessels and fibroblastic and osteoblastic activities were detected (Fig. 4T6). At 4-week intervals, the PDL and the woven bone showed the same changes as the previous intervals, plus large cementocytes inside the root cementum were detected (Fig. 4T8). Different regions of the alveolar bone showed extravasated blood vessels compared to the control group.

## Discussion

Based on previous literature, this is the first trial assessing the utilization of CGF injection for accelerating orthodontic tooth movement.

New Zealand white rabbits were selected as an animal model for this study as they are considered one of the most suitable animals for getting a clear image of bone alterations under stress [39]. Furthermore, the bone turnover in the rabbits is rapid more than in other species [40].

CGF contains many growth factors; hence, it has been proven that they are key elements for bone cell recruitment, activation, proliferation, differentiation, and survival. CGF was utilized in this experimental study [25, 41, 42].

In this investigation, CGF was injected submucosally buccal and palatal opposite the maxillary first molars on the experimental sides. Submucosal application is the predominant approach for administering local pharmacological agents to accelerate tooth movement [7, 43]. The injection technique and amount were similar to the previous study, which utilized PRP for the acceleration of OTM [36].

NiTi coil springs were chosen because they do not display quick force decay, produce a steady light force that has been testified to be beneficial in space closing, and give superior oral health compared to elastomeric chains [44, 45].

During the first week, nonsignificant differences were observed between the two groups. This phenomenon might likely be attributed to the earliest stage of orthodontic treatment, which occurs within the PDL space and may persist for approximately one week before any more tooth movement becomes noticeable [46]. Conversely, a significant increase in tooth movement was observed during the second, third, and fourth weeks following CGF injection, compared to the control sides (*p* = 0.003, 0.001, 0.002; respectively). These results indicate a clear association between CGF injection and the hastening of orthodontic tooth movement. This was following the results obtained by previous animal studies utilizing PRP injection [30, 47].

The study found that the rate of tooth movement increased by approximately 1.5 times after the injection of CGF, compared to the control group. The acceleration seen in this study was comparable to the acceleration achieved after PRP injection as reported by Alaa et al. [30], and after the administration of moderate and high concentration PRP as reported by Gulec et al. [37]. In contrast, Rashid et al. [47] observed a 2.13-fold increase in acceleration on the PRP side, which is greater than the findings of this investigation. The possible cause of this could be attributed to variations in their injection method (intraligamental and submucosal) and the higher frequency of injections employed in their research. Histologically, the rate of tooth movement can be controlled by bone remodeling, bone formation at the tension side, and bone resorption at the compression side during tooth movement [48]. Our results were consistent with Choi et al. [49], who found that bone remodeling in rats jumps after the first week, with the activity continuing till the start of the third week. Moreover, these outcomes agree with those of Pavlin et al. [50], where there was evidence of new bone formation from the first week to day 12. The appearance of irregularly arranged large osteocytes occupying the woven bone of the socket at the tension side of both groups appeared earlier in the CGF group. This could be clarified by the ability of the CGF to enhance osteogenic differentiation and mineralization of the cranial defect model of rats in vivo [50]. Also, bone resorption on the compression side appeared earlier in the CGF group than in the control group. The increased rate of bone resorption could have been explained by the existence of CGF several growth factors that induced osteoblastic and osteoclastic activity [41]. On the compression side, the clinical finding is verified. The PDL fibers were arranged irregularly in the two groups but to a lesser extent in the CGF group. This was mainly the result of the compression effect from the tooth to the PDL, which would finally followed by tooth movement through a complex process of molecular and cellular routes [51]. Moreover, more widened blood vessels were observed in PDL in the CGF group than in the control group. This was most likely brought on by the inflammatory mediators that the PDL tissues released secondary to the mechanical effect of orthodontic treatment and those initially present within the CGF as vascular endothelial growth factor (VEGF), a potent vascular dilator [52, 53].

### Study limitations and clinical significances

Our study had some limitations; the long-term evaluation, the efficacy of different doses, and injection frequency are critical and may have a significant impact on clinical implementation of this new technique. Second, due to anatomical differences, the influence on mandibular teeth must be explored in future investigations. However, under the current study settings, the results could be clinically significant for orthodontic practice. CGF could be utilized as a simple, safe, and cost-effective method for accelerating orthodontic tooth movement.

## Conclusion

Within the limitation of this study, it could be concluded that CGF has the potential to effectively enhance orthodontic tooth movement without adverse clinical or histological effects.

## Data Availability

On reasonable request, the datasets utilized and analyzed during the present study are accessible from the corresponding author.

## Abbreviations

BDGF: Brain-derived growth factor
CGF: Concentrated Growth Factor
FGF: Fibroblast growth factor
H&E: Hematoxylin and eosin
IGF: Insulin-like growth factor
OTM: Orthodontic tooth movement
PCs: Platelet concentrates
PDGF: Platelet-derived growth factor
PRF: Platelet-rich fibrin
PRP: Platelet-rich plasma
TGF-β1: Transforming growth factor-β1
TGF-β2: Transforming growth factor-β2
VEGF: Vascular endothelial growth factor
